# Effectiveness of a new model of primary care management on knee pain and function in patients with knee osteoarthritis: Protocol for THE PARTNER STUDY

**DOI:** 10.1186/s12891-018-2048-0

**Published:** 2018-04-30

**Authors:** David J. Hunter, Rana S. Hinman, Jocelyn L. Bowden, Thorlene Egerton, Andrew M. Briggs, Stephen J. Bunker, Jessica Kasza, Andrew B. Forbes, Simon D. French, Marie Pirotta, Deborah J. Schofield, Nicholas A. Zwar, Kim L. Bennell, the PARTNER Study Team, Karen Shuck, Charlotte Marshall, Michelle King, Anna Wood

**Affiliations:** 1 Institute of Bone and Joint Research, Kolling Institute, University of Sydney, Sydney, Australia; 2 Department of Rheumatology, Royal North Shore Hospital, Sydney, NSW Australia; 3 Centre for Health, Exercise and Sports Medicine, Department of Physiotherapy, The University of Melbourne, Melbourne, Victoria Australia; 4 School of Physiotherapy and Exercise Science, Curtin University, Perth, WA Australia; 5 Department of Physiotherapy, The University of Melbourne, Melbourne, Victoria Australia; 6 Biostatistics Unit, School of Public Health and Preventive Medicine, Monash University, Melbourne, Victoria Australia; 7 School of Rehabilitation Therapy, Queen’s University, Kingston, Ontario Canada; 8 Department of Chiropractic, Faculty of Science and Engineering, Macquarie University, Sydney, NSW Australia; 9 Department of General Practice, The University of Melbourne, Melbourne, Victoria Australia; 10 Department of Economics, Faculty of Business and Economics, Macquarie University, Sydney, NSW 2109 Australia; 11 School of Public Health and Community Medicine, University of New South Wales, Sydney, NSW Australia; 12 School of Medicine, University of Wollongong, Wollongong, NSW Australia; 13 Centre for Health, Exercise and Sports Medicine, Department of Physiotherapy, The University of Melbourne, Melbourne, Victoria Australia

**Keywords:** Primary care, Knee osteoarthritis, Model of service delivery, RCT, Clinical trial

## Abstract

**Background:**

To increase the uptake of key clinical recommendations for non-surgical management of knee osteoarthritis (OA) and improve patient outcomes, we developed a new model of service delivery (PARTNER model) and an intervention to implement the model in the Australian primary care setting. We will evaluate the effectiveness and cost-effectiveness of this model compared to usual general practice care.

**Methods:**

We will conduct a mixed-methods study, including a two-arm, cluster randomised controlled trial, with quantitative, qualitative and economic evaluations. We will recruit 44 general practices and 572 patients with knee OA in urban and regional practices in Victoria and New South Wales. The interventions will target both general practitioners (GPs) and their patients at the practice level. Practices will be randomised at a 1:1 ratio. Patients will be recruited if they are aged ≥45 years and have experienced knee pain ≥4/10 on a numerical rating scale for more than three months. Outcomes are self-reported, patient-level validated measures with the primary outcomes being change in pain and function at 12 months. Secondary outcomes will be assessed at 6 and 12 months. The implementation intervention will support and provide education to intervention group GPs to deliver effective management for patients with knee OA using tailored online training and electronic medical record support. Participants with knee OA will have an initial GP visit to confirm their diagnosis and receive management according to GP intervention or control group allocation. As part of the intervention group GP management, participants with knee OA will be referred to a centralised multidisciplinary service: the PARTNER Care Support Team (CST). The CST will be trained in behaviour change support and evidence-based knee OA management. They will work with patients to develop a collaborative action plan focussed on key self-management behaviours, and communicate with the patients’ GPs. Patients receiving care by intervention group GPs will receive tailored OA educational materials, a leg muscle strengthening program, and access to a weight-loss program as appropriate and agreed. GPs in the control group will receive no additional training and their patients will receive usual care.

**Discussion:**

This project aims to address a major evidence-to-practice gap in primary care management of OA by evaluating a new service delivery model implemented with an intervention targeting GP practice behaviours to improve the health of people with knee OA.

**Trial Registration:**

Australian New Zealand Clinical Trials Registry: ACTRN12617001595303, date of registration 1/12/2017.

## Background

Arthritis and musculoskeletal conditions are more prevalent in Australia than any other National Health Priority area, including cancer, diabetes and obesity [1]. Osteoarthritis (OA) in particular is a leading cause of pain, disability and early exit from the workforce in Australia [2], with the knee commonly affected. Arthritis leads to a substantial loss of income [3] (with a resultant increase in welfare dependency) [4] and reduction in taxation revenue, and a significantly increased risk of falling into poverty [5].

Current care of people with OA in Australia is inconsistent with clinical guidelines, with 57% of people not receiving appropriate care according to evidence-based quality indicators [6]. As a result, 68% of Australians with arthritis report “doing badly” or “fairly badly” with respect to how their lives are affected by arthritis [7]. Knee OA in Australia is mostly managed in general medical practice. A recent systematic review has highlighted that general practitioners (GPs) are hampered in their treatment of this chronic condition by lack of knowledge of non-surgical management options, and limited access to services that support the key recommended options such as lifestyle and behavioural changes [8]. To address this gap, GPs and other health professionals [9–11] have called for new models for OA primary care that provide clear clinical pathways and support networks to allow multi-disciplinary input and lifestyle counselling for ongoing self-management of OA. This trial aims to address a major evidence-to-practice gap in primary care management of OA by evaluating a new service delivery model implemented with an intervention targeting GP practice behaviours to improve the health of people with knee OA. Importantly, the model of service delivery aligns with key recommendations of established models of care in Australia [10] and will provide important policy-relevant data to support implementation and scalability of these models.

Current clinical guidelines emphasise non-surgical treatments, coupled with appropriate pharmacologic care, as the cornerstone of OA management [12, 13]. In particular, education and advice, exercise and physical activity, and weight management are the gold standards. Benefits of exercise were well-established in a 2015 Cochrane Review [14] with effect sizes higher than, or comparable to, those of simple analgesics and oral non-steroidal anti-inflammatories [15]. Patients with knee OA often report preferring exercise over drug treatments [16], due to a lower risk profile. For those who are overweight or obese, weight loss is critical to improving overall health and joint symptoms [17]. Meta-analysis suggests patients should reduce body weight by at least 5% to gain improvement in pain and function [18], while a large RCT [19] has provided further evidence of the benefits of ~ 10% weight loss in OA populations, particularly when diet is combined with exercise.

Analysis of Australian BEACH data [20] from 487,000 GP consultations for OA found rates of drug prescription were much higher than rates of lifestyle management (79 vs 21/100 knee OA contacts). Most referrals were directly to orthopaedic surgeons (68%) with few to physiotherapists (18%). Among people with hip/knee OA referred for orthopaedic management at a major Australian tertiary hospital, 80% felt they had not been sufficiently educated about OA and 33% had not engaged in any core non-drug conservative treatment [21]. A meta-analysis assessing OA care [22] found that quality indicator pass rates were suboptimal particularly for non-drug, non-surgical treatment, demonstrating that this is a worldwide problem. Such gaps in care are highly relevant as poorer access to adequate information about OA, poorer perceived quality of care and poorer perceived GP knowledge about treatment options are associated with worse patient outcomes [7].

Undertaking regular exercise and losing weight is difficult for many people with knee OA and requires long-term behaviour change, coupled with appropriate support. A scoping review highlighted many barriers to undertaking exercise, including lack of knowledge and/or incorrect beliefs about capabilities and consequences [23]. Similarly, 89% of obese patients with knee OA consider lack of motivation to be their greatest barrier to weight loss [24]. Effective communication and support from health professionals are vital for self-belief and sustained motivation [25]. However, many clinicians typically practice within a biomedical framework that inadequately considers psychosocial factors that are important in disease control [26]. In addition, time constraints in consultations and a lack of knowledge, skills and confidence in behavioural counselling are reported as barriers to optimal OA care delivery by GPs [8].

The need for new, effective primary care models was identified as the research priority most likely to alleviate Australian OA burden at the 2012 Australian OA Summit [27]. It was also identified in a White Paper by Arthritis Australia following stakeholder consultation. [28] There is also a wealth of evidence to support the system-level benefits of the development and evaluation of models of care and their models of service delivery [29], including a broad acceptance of this approach in Australia [30]. As such we have performed extensive work to develop a new model of service delivery: the PARTNER model, and have designed an implementation plan for delivering the model in the current Australian primary care context. PARTNER is underpinned by the Chronic Care model [31], evidence-based clinical practice guidelines [12, 13], and informed by broad stakeholder input (consumers, GPs, physiotherapists, rheumatologists, nurses, behaviour change experts, policy makers, health insurers and consumer advocates) and the knowledge and experience of the Osteoarthritis Healthy Weight For Life Program (OAHWFL) [32]. The implementation plan was designed using the ‘Behaviour Change Wheel’ and informed by the Theoretical Domains Framework [33]. The implementation plan targets GPs via GP professional development modules and provision of a desktop EMR support tool, and the PARTNER model targets their patients who are referred to a centralised, remotely-delivered, multi-disciplinary Care Support Team (CST) for proven exercise, weight loss and pain management interventions. Further details on the theory underpinning the development of the PARTNER model of service delivery and the implementation plan will be presented in a separate paper.

The aim of this project is to implement the PARTNER model for people with knee OA in an Australian primary care setting and to evaluate the effectiveness and cost-effectiveness of the PARTNER model compared with usual care. We hypothesise the PARTNER model will be superior. We will also conduct a process evaluation to assess success of the implementation plan, PARTNER model fidelity, identify contextual influences on scalability and sustainability and identify cost considerations for scaling up the GP-level intervention and CST service throughout Australian primary care.

## Methods/ design

### Trial design

The PARTNER trial is a mixed-methods study including a two-arm, pragmatic, cluster randomised controlled trial (RCT), a health economic analysis and nested qualitative evaluations. The primary endpoint for analysis is at 12 months. The protocol conforms to the Standard Protocol Items Recommendations for Interventional Trials (SPIRIT) 2013 Statement for developing clinical trial protocols [34]. The trial was prospectively registered on the Australian New Zealand Clinical Trials Registry (ACTRN12617001595303), and the World Health Organisation Universal Trial Number is U1111–1197-4809.

### Study population and setting

We will recruit 44 general practices with a minimum of two GP participants per practice, and 572 of their patients.

#### General practices and GPs

will be recruited from metropolitan and regional areas of Victoria and New South Wales, Australia, through our GP Research Networks. Practice elibility criteria include: i) at least two registered GPs at a practice agree to be involved; ii) use of a general practice clinical desktop system compatible with the electronic desktop IT support tool being used for the study (cdmNet); iii) current public liability insurance; iv) consent to be randomised, and v) did not participate in the pilot study. GPs are eligible if they: i) work in a participating practice; ii) are registered with professional indemnity insurance; iii) are willing to undertake the study as per the protocol, and iv) currently treat patients with knee OA.

#### Patients

Potential patients will be identified from the general practice’s patient database, and the inclusion criteria are based on the National Institute for Health and Care Excellence (OARSI) Guidelines for Osteoarthritis Care and Management in Adults [35]. Eligible patient participants will be i) aged 45 years or older; ii) report activity-related knee pain for more than 3 months; iii) report knee pain over the past week as greater than 3 on an 11-point numerical rating scale (NRS, with terminal descriptors of “no pain” and “worst possible pain”) at screening, and iv) either be a patient of a GP participant or agree to see a GP participant in the same practice. Patients are not eligible to participate if they cannot provide informed consent in English, have limited mobility requiring the use of a wheelchair or scooter or are non-ambulatory, have had or are booked in for knee replacement surgery in the knee they are seeking treatment for, are terminally ill, have rheumatoid arthritis or gout or other severe inflammatory condition, or are undergoing treatment for serious medical/psychiatric conditions that would preclude participation (e.g. cancer treatment).

### Trial procedures

**Fig. 1:**
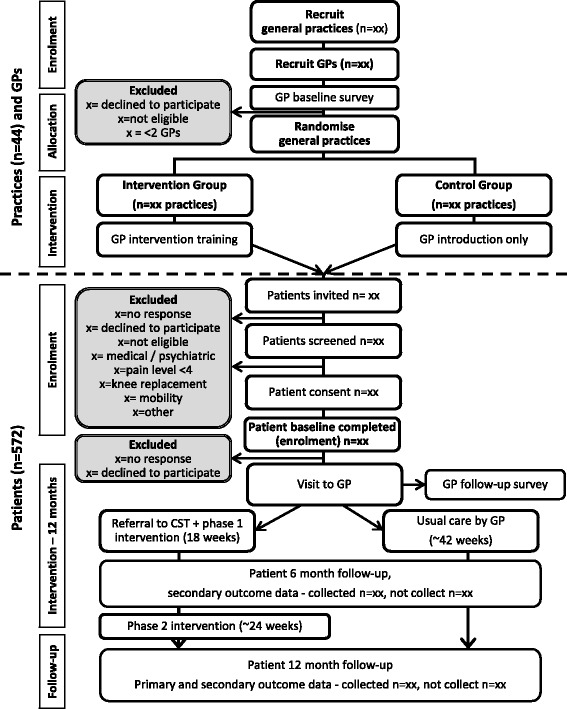
Flow diagram of the study. The recruitment of patients will occur after the practices and GPs have been recruited and randomised, and after GPs have completed their baseline assessment. Patients will only be considered to be enrolled in the trial after they have submitted both their informed consent forms and completed the baseline assessment

Figure 1 outlines the trial phases. General practices, GPs and patients will be screened and consented as outlined below. GPs will complete surveys at baseline and after all their participants have had an initial consultation. Patients will complete assessments at baseline, 6 months and 12 months, and after their initial GP consultation. An additional survey will be sent to all patients at 3 months to assess participation in study components, and monthly surveys to collect health service usage data for economic evaluation. Surveys will be sent and completed via the Research Electronic Data Capture tool (REDCap) [36] online database software, or if necessary via hard copy surveys sent and returned by mail.

#### Recruitment

General practices and GPs will be recruited through the Victorian Primary Care Practice-Based Research Network (VicReN, based at the University of Melbourne, Victoria, Australia) and the New South Wales (NSW) Primary Health Care Research Network (PHReNet, based at University of NSW Sydney, Australia). At least one phone call and practice visit will be undertaken to provide a detailed description of the study, screen the practices and GPs for inclusion, gain informed consent, and to familiarise GPs regarding the required training in study methods, the professional development obligations if allocated to the intervention group (see GP intervention below), and the support provided by the study team.

Patients will be recruited from the practice’s patient database. Practice staff will identify patients ≥45 years who are patients of the enrolled GPs. The list will be randomised for the mail out, and before the practice is advised of its group allocation GPs will have the opportunity to exclude any patients they know do not meet the inclusion criteria. The practice will send out letters inviting participation on behalf of the researchers, using the practice’s usual method of communication with their patients: post or email. Invitation letters will be sent out in batches until the required number of patients from that practice are recruited. The exclusion list will be checked by the PARTNER Study GP coordinators prior to the letters being sent to ensure all eligible participants in each batch are contacted. Interested patients will complete an online screening questionnaire. Those who pass online screening will be contacted by trial staff for further phone-based confirmation of eligibility and to discuss details of study participation. Eligible patients will be mailed a participant information statement and consent form for participation in the study, and separate consent form authorising the study to access their complete Medicare Benefits Schedule (MBS) and Pharmaceutical Benefits Scheme (PBS) data for the economic evaluation.

All recruited general practices, GPs and patients will provide written informed consent. This study involves limited disclosure for patients so as to limit pre-conceived ideas as to the superiority of one service delivery method. The information and consent documents provide information about the purposes, potential risks and processes of the study, but do not include specific details of the intervention. Consented patients will receive an email link to, or mailed hard copy of, the baseline survey for completion. Once the baseline survey has been completed and checked by the research team the participant is deemed to have entered the study. The 12-month patient intervention commences at this time (Fig. 1).

#### Randomisation and allocation concealment

General practices will be randomised, rather than individual GPs or patients, as the intervention involves changes at the practice level. Once GP recruitment at the site is completed, general practices will be randomly allocated to either the intervention or usual care control group at a 1:1 ratio, and by random permuted blocks of sizes 8, 10 and 12. Stratification will be by practice size (< 4 GPs, ≥4 GPs) and location (metropolitan, regional/rural), based on the Australian Statistical Geography Standard (ASGS) Remoteness Structure (2011) [37]. Metropolitan areas will correspond to the ASGS major cities classification (RA1), while regional/rural will be a combination of the inner regional (RA2), outer regional (RA3), remote (RA4) and very remote classifications (RA5). Offsite computer-generated randomisation will be conducted by the study statistician. Opaque, sealed envelopes will be used to conceal allocation and are kept in a locked location.

Patient participants are considered ‘assessors’ in this study as all primary outcomes are self-reported. Thus, as participants are blinded to group allocation the trial is also ‘assessor’ blinded. Statistical analyses will be undertaken by blinded statisticians. Staff of VicReN and PHReNet assigned to assist each practice will be blinded until the point of practice allocation. Research staff involved in patient screening will be blinded until after they have made the eligibility decision. It is not possible to blind the GPs to group allocation, however, they are requested not to discuss the study allocations with their patients.

### Intervention

**Fig. 2:**
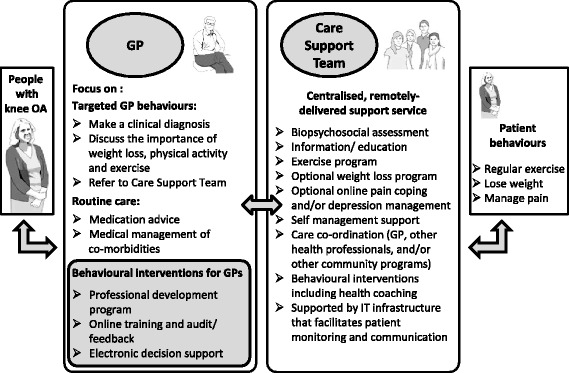
PARTNER model of service delivery

The PARTNER model includes management by both the GP and the new CST service to improve patient outcomes (Fig. 2). The implementation intervention aims to facilitate the GPs’ role in the PARTNER model. Patient participants will all have an initial visit to their GP for confirmation of an OA diagnosis and a knee OA focused consultation as their GP sees fit. Patient participants of GPs allocated to the intervention group will receive care described in the PARTNER model. Delivery of the PARTNER model depends on GPs receiving the implementation intervention (GP intervention) and the patient receiving care for 12 months by the CST service. These two components are described below. GPs in the control group will receive no intervention and their patients will not have access to the CST. After the initial GP consultation, control patient participants will continue to receive ‘usual GP care’ for their knee OA.

#### GP intervention

According to the PARTNER model, GP management includes confirmation of OA diagnosis, providing advice on the importance of exercise and weight management, reviewing their pain medications, referring to the PARTNER CST and if appropriate preparing a Chronic Disease Management Plan. The GPs allocated to the intervention group will be required to undertake a range of “core” professional development activities related to best practice management of knee OA to facilitate their understanding of the PARTNER model before seeing patients in the study. All professional development activities can be completed online and are self-paced. The topics are based on the NICE Clinical Guidelines for the management of OA [12], and include:

- *GP self-audit activity*: A self-audit and feedback activity will guide GPs to reflect on their management of recent patients with OA and self-identify any areas for improvement. Part 1 of the audit is a required element of the study and should be completed before the GP completes any other professional development activities. GPs will be asked to identify and describe five recent consultations where they diagnosed and managed a person presenting with chronic knee pain. The GPs will be asked to answer 20 questions on the priorities for best practice diagnosis and management of knee OA. The questions were selected and modified for the audit from three sources by an expert panel [38–40]. Based on their score, the GPs will then receive feedback consisting of short evidence summaries that address potential areas to improve best practice care.
  Part 2 of the audit is optional and completed after all their patients have completed their involvement with the study. GPs are asked to repeat the 20 questions from Part 1.
  They are provided with their scores from both parts of the audit and given the opportunity to revisit the priority components of care for knee OA. Completion of both parts of the audit allows GPs to claim Quality Improvement and Continuing Professional Development (QI&CPD) category 1 points from the Royal Australian College of General Practitioners (RACGP).
- *Knee osteoarthritis in general practice’ online learning module:* An on-line professional development module on evidence-based knee OA diagnosis and treatment developed in collaboration with the RACGP. The approximately 1-h package covers current best-practice recommendations for non-surgical management of OA. The module is located on the RACGP website as part of their QI&CPD program [41]. Completion of it confers one RACGP QI&CPD point (category 2).
- *‘Introduction to the PARTNER Study*: GPs will be asked to view a short video on the PARTNER study, which outlines the aims of the study and will briefly introduce the PARTNER model (Fig. 2). It gives an overview of the intervention processes and tasks that need to be completed by both the GP and the patients enrolled in the study, including study aims, patient recruitment, study tasks for patients and GPs, governance requirements and reporting serious adverse events and study contact details.

In addition to the professional development program, GPs are provided with desktop OA management tools via cdmNet software. cdmNet is a network of online computing services and infrastructure designed to help GPs and other healthcare providers manage people with chronic diseases, and facilitate referral to other health professionals [42]. cdmNET includes decision support features to prompt GPs in evidence-based OA consultation activities, an OA care plan, a mechanism for referral to the CST and HWFL, and PARTNER-specific educational resources which can be printed out for the patient. All general practices and GPs allocated to the intervention group will receive training in the cdmNet software, and free copies of the software if required.

Optional skills and capacity building modules are also available online for GPs who would like further training on specific topics. Topics include: how lifestyle changes can improve symptoms and improve function; different options for non-surgical management of OA; how to have conversations with patients about OA, weight loss and exercise; brief advice incorporating a motivational interviewing approach; and the importance of optimism and positive expectations on outcomes. Further reading and links to other OA resources and research will also be provided for interested GPs.

#### The PARTNER care support team

The PARTNER CST is a centralised, multidisciplinary team of health professionals trained in best-practice OA management, health coaching and behavioural change. The CST will support patients to manage their knee OA and will help the GP facilitate additional healthcare services, if required. After referral, a member of the CST will contact the participant patient to discuss the CST service, the different OA management options, help the patient prioritise their needs and agree goals.

The CST will be comprised of a multidisciplinary allied health team with nine members from a range of professions including physiotherapy, exercise physiology and occupational therapy. The CST will be trained in behaviour change methodology and health coaching by our partner, HealthChange Australia. The CST training will comprise 2 workshops (total 3 days) on the HealthChange Australia™ methodology [43]. Between the 2 workshops the CST members will each be required to undertake two practice phone calls to four patients, and to self-appraise their skills for two of these patients. The calls will be recorded, and one pair of calls from each member sent to HealthChange Australia for review and group feedback. Additional webinar training sessions for the CST will be held on best practice management of knee OA, the PARTNER study procedures and the software used for the study.

The CST will deliver the intervention remotely via phone, video call, email, post and/or SMS contact, as per individual patient preference. The patient’s first contact session with the CST will occur at approximately one month from patient enrolment, contingent on GP availability. During the first contact session a member of the CST will undertake a biopsychosocial assessment of the patient and provide further education about OA and different management options. A tailored care plan aligned to the patient’s individual health needs will be developed with a particular focus on the study’s priority areas of weight loss, exercise and physical activity. Secondary intervention options for other issues are also available for patients to undertake (see *Secondary interventions* below). Patient priorities and goals should evolve over the course of the intervention. The CST intervention will occur in two phases (Fig. 1):

1. Phase 1 – first 18 weeks. The patient will be contacted once per fortnight on average, or as agreed with the patient. The CST will provide ongoing self-management advice and support, to address the goals and activities of their tailored care plan.

2. Phase 2–6 month maintenance period (or until the end of the 12-month intervention period). Patients will be encouraged and supported to develop strategies to self-manage their OA. They will be contacted monthly, or as agreed, to monitor their progress and address any new or ongoing issues as required. Patients can still be re-referred to their GP or other allied health professionals if an escalation of care is required. However, patients cannot elect to participate in the HWFL weight-loss program during this phase.

The CST will provide updates to the GPs on the progress of their referred patients via cdmNET at three time points (after initial consultation, after 18 weeks and at the end of the patient’s involvement with the CST), or on request by the GP. The reports will detail any strategies and actions agreed with the patient, and their progress to date. The CST will refer the patient back to their GP if they require a medication review, or if they have any other medical condition that affects participation in the trial or an escalation of care. If the situation is urgent the CST member will ring the GP to discuss. If the situation is routine the CST will correspond with the GP via cdmNET.

#### Priority interventions

All patients allocated to the Intervention group will be offered the ‘priority’ interventions. These interventions are recommended by the NICE Clinical Guidelines for the management of all people with knee OA [12]. While patients are strongly encouraged to undertake all the priority interventions recommended by the CST, it is ultimately their decision on the interventions to undertake. The priority interventions include:

- *Education:* provision of OA educational resources compiled by the PARTNER team and our partner organisations, as well as links to Arthritis Australia’s OA consumer website ‘*myjointpain.org.au*’ and the ‘pain*HEALTH*’ website, [44, 45] a brochure introducing the CST and a range of other information sources and other self-monitoring tools.

b. *Muscle strengthening program:* A physiotherapist-developed home exercise program for leg strengthening which includes the common exercises used to strengthen the thigh and buttock muscles, and provides tips for progressing the exercises and how to stay motivated with an exercise program. Patients will be provided with exercise resistance bands to assist them to progress. This program will be provided by the CST.

c. *Physical activity plan:* the CST will help the patient with strategies to increase their incidental and general physical activity levels.

d. *Weight loss advice and support*: If the patient has a BMI ≥27 kg/m^2^, the patient will be offered the option of participating in the remotely-delivered “Healthy Weight for Life®” weight loss program which has been shown to be effective in people with OA [32]. The program is an 18-week, three-phase approach provided by PRIMA Healthcare, and involves a very low-calorie diet (KicStart™), a portion-controlled eating system, an exercise program, and educational resources on a healthy diet and lifestyle. After the 18-weeks patients will be referred back to the PARTNER CST, and if required can continue to be provided with weight loss advice until the end of the intervention period. Patients who do not wish to undertake the HWFL program, or are not eligible for the program, will continue to be managed by the CST throughout the whole intervention period, as described above. They will be offered all other relevant components of the intervention.

e. *Medication review:* Patients will be offered a review of their current OA medications. This should be undertaken by the GP at the initial consultation, however their medications will continue to be discussed by the CST, using an algorithm previously developed for Arthritis Australia’s *myjointpain.org.au* website*.* Patients will be encouraged to speak to their GP or local pharmacist if they require a medication review.

#### Secondary interventions, online management tools and community run services

Patients may also be directed to one or more online tools, or community run services for the management of different aspects of their OA. The online tools have been developed by experts in their field. Referral to these online services will be offered if the patient meets the pre-determined criteria for stepping up care and/or has identified as their priority for action. These components are *optional* for the patient and include:

- Advice on mobility aides or secondary therapies such as walking sticks, orthoses, or heat pads.
- *Pain coping skills training*: an online 8-week Cognitive Behavioural Therapy (CBT) program for pain coping skills training which has been shown to be effective in knee OA [46]. The pain coping skills training program is an online program comprised of eight modules that provide interactive training in cognitive or behavioural pain coping skills. It is being provided free of charge for patients in the study.
- *Depression - This Way Up*: an online CBT program for education and treatment of anxiety and depression [47]. It will be offered to intervention patients in the study free of charge [48].
- *Insomnia and sleep – SHUTi*: the online CBT program “SHUTi” was designed to help people with insomnia identify and change thoughts or behaviours that influence sleep patterns [49]. The course is offered in Australia through the Black Dog Institute [50]. The completion of this course is optional and would be at an additional cost.
- *Community-based services and facilities*: Depending on the patient’s preference for service delivery (e.g. group vs individual), they may be directed to services or facilities run by community groups or other commercial entities, such as exercise classes, public gyms or other sporting organisations. These options would be at an additional cost.
- *Work productivity*: If patients self-report a decrease in their work productivity due to their knee OA they will be given the option of being referred to a vocational counsellor in their area. This option would be at an additional cost.

### Usual care

Patients in the control group will be asked to visit their GP for an initial consultation. GPs will not refer their patients to the CST, but will manage the patient’s OA in their usual manner (usual care). Management of the patient’s OA is purely at the discretion of the GP. GPs will only receive a brief introduction to the study. GPs may refer to any third-party provider, which may incur additional out-of-pocket expenses. Patients may attend these services at their own discretion and cost. Once the GP in this group has completed their involvement in the study (i.e. all of their patients have had their final follow-up assessment), they will be offered the opportunity to undertake the training and professional development opportunities offered to the GPs in the intervention group. The exception would be the provision of the cdmNet software and training.

### Outcome measures

Table 1 summarises the primary and secondary outcome measures. The outcomes include patient-level validated measures recommended for OA by the Osteoarthritis Research Society International (OARSI) and International Consortium for Health Outcomes (ICHOM) [51, 52], or have been used in other similar trials. Patients will be asked to nominate their most symptomatic knee (provided it has not undergone arthroplasty), which will be considered their “study knee” for the duration of the trial. Primary and secondary outcome data will be collected at baseline, 6 and 12 months after enrolment and are self-reported unless otherwise specified in Table 1. 24-month follow-ups will also be conducted. The two primary outcomes are:

1. Change in average knee pain at 12 months: overall average pain over the past week self-reported via an 11-point NRS with terminal descriptors of ‘no pain’ (score 0) and ‘worst pain possible’ (score 10).

**Table 1:** Summary of primary and secondary data collected from study patient participants to determine intervention effectiveness

Outcome	Measurement tool
Primary Outcome Measures	
Knee pain	11-point numerical scale
Knee function	Knee injury and Osteoarthritis Outcome Score (KOOS) - function in daily living subscale
Secondary Outcome Measures	
Knee pain	KOOS pain subscale
Knee function	KOOS - function in daily living subscale
Knee OA symptoms	KOOS - other symptoms subscale
Knee function with sport and recreation	KOOS - function with sport and recreation subscale
Knee quality of life	KOOS - knee-related Quality of Life subscale
Weight and BMI (calculated)	Self-report
Health-related quality of life	Assessment of Quality of Life (AQoL-8D)
Depression	Patient Health Questionnaire (PHQ 9)
Sleep impairment	PROMIS adult Sleep-Related Impairment Short Form 8a
Fatigue	PROMIS Fatigue Short Form 8a
Global rating of change	7-point numerical rating scale
Satisfaction, overall with treatment	7-point numerical rating scale
Satisfaction with change in symptoms	7-point numerical rating scale
Work productivity	Work Productivity and Activity Impairment Questionnaire: Osteoarthritis of the Knee V2.0 (WPAI:OA)
Health Care Expenditure	Medicare Benefits Schedule (MBS) and Pharmaceutical Benefits Scheme (PBS) data.
Economic evaluation of the cost-effectiveness of the intervention	Based on the Assessment of Quality of Life (AQoL-8D)

2. Change in physical function of the knee at 12 months: knee function will be measured using the function in daily living subscale of the Knee injury and Osteoarthritis Outcome Score (KOOS) [53]. The KOOS questionnaire measures symptoms and functional limitations associated with knee OA using patient-reported outcome measures. The questionnaire consists of five subscales; pain, other symptoms, function in daily living (ADL), function in sport and recreation (Sport/Rec) and knee-related Quality of Life (QOL), measured using Likert responses scored from 0 to 4. The questions pertain to the previous seven days. The KOOS is a widely used disease-specific instrument whose validity, reliability and responsiveness have been demonstrated in a range of OA studies [54].

Our secondary outcome measures are:

1. Change in knee pain at 6 months: overall average pain over the past week will be self-reported via a NRS and the pain sub-scale of the KOOS (as described above).
2. Change in physical function of the knee at 6 months: will be measured using the *Function (Activities of Daily Living)* subscale of the KOOS.

3. Change in other OA symptoms of the knee at 6 and 12 months will be measured using the *Symptoms* subscale of the KOOS.

4. Change in QoL at 6 and 12 months will be measured using the *Quality of Life* subscale of the KOOS.

5. Change in function of the study knee during sport and recreational activities at 6 and 12 months will be measured using the *Function (sports and recreational activities)* subscale of the KOOS.
6. Change in weight and BMI at 6 and 12 months. BMI will be calculated using baseline height data. Patients will be asked to use the same set of scales to measure their weight for all time points. It is recommended that these outcomes are assessed for all OA clinical trials that target symptom-modifying interventions [51].
7. Change in health-related QoL at 6 and 12 months will be assessed via the *Assessment of Quality of Life* (AQoL-8D) [55]. The AQoL-8D is a health-related multi-attribute utility quality of life instrument, initially designed for use in economic evaluation studies. This instrument has 35 items in 8 separately scored dimensions; independent living, relationships, mental health, coping, pain, senses, self-worth and happiness. The questions pertain to the previous 7 days.
8. Change in depression (mood) at 6 and 12 months will be measured using the *Patient Health Questionnaire* (PHQ9) [56]. The PHQ9 is a multi-purpose instrument for screening, diagnosing, monitoring and measuring the severity of depression. It is used widely for identifying depression in chronic conditions including OA. There are nine questions scored on a 4-point scale (0 = not at all, 3 = nearly every day). The questions pertain to the previous 14 days. Score responses are *normal*, *mild*, *moderate*, *moderately severe, severe* and *very severe*. Any patient with a classification of *severe* or above, or presents with thoughts of self-harm, will be referred back to their GP for their follow-up.
9. Sleep impairment at 6 and 12 months will be measured using the *Patient-Reported Outcomes Measurement Information System* (PROMIS™) adult Sleep-Related Impairment Short Form 8a (patient self-report) [57]. The tool consists of eight questions that ask patients to rate their perceptions of alertness, sleepiness, and tiredness during usual waking hours, and the perceived functional impairments during wakefulness associated with sleep problems or impaired alertness. The questions pertain to the previous seven days. Five response options are used ranging in value from 1 (not to all) to 5 (very much). The total raw score is calculated by the sum of values of the response to each question (min 8, max 40).
10. Change in fatigue at 6 and 12 months will be measured using the *PROMIS*™ Short Form 8a (patient self-report) [57]. The tool consists of eight questions that ask patients to rate their perceptions of fatigue symptoms ranging from mild subjective feelings of tiredness to an overwhelming, debilitating, and sustained sense of exhaustion that likely decreases one’s ability to execute daily activities and function normally in family or social roles. Fatigue is divided into the experience of fatigue (frequency, duration, and intensity) and the impact of fatigue on physical, mental, and social activities over a 7-day period. Five response options are used ranging in value from 1 (not to all) to 5 (very much). The total raw score is calculated by the sum of values of the response to each question (min 8, max 40).
11. Change in patient’s global rating of change in knee OA at 6 and 12 months. Patients will be asked to rate their overall perceived change in OA on a 7-point scale ranging from “much worse” to “much better”.
12. Satisfaction with treatment will be measured at 6 and 12 months on a 7-point scale ranging from “extremely unsatisfied” to “extremely satisfied”.(47)

13. Satisfaction with change in knee OA symptoms (outcome) will be measured at 6 and 12 months on a 7-point scale ranging from “extremely unsatisfied” to “extremely satisfied” [58].

14. Change in lost productivity at 6 and 12 months will be measured using the *Work Productivity and Activity Impairment Questionnaire*: Osteoarthritis of the Knee V2.0 (WPAI:OA) [59]. This instrument measures the time away from work because of knee OA and the effect of knee OA on productivity while at work. The questions pertain to the previous seven days.

15. Healthcare expenditure will be extracted from the MBS and PBS data. Medicare data includes information on medical visits and procedures, and the associated costs and PBS data includes information on prescription medicines filled at pharmacies. We will seek patients’ consent to access their MBS and PBS data from the Australian Government Department of Human Services (DHS, approval no. MI7185). A structured health diary will be used to collect other health services on the use of services not included in MBS and PBS data such as hospitalisation, non MBS-funded allied health service use and over the counter medications.
16. Economic evaluation of the cost effectiveness of the intervention.

#### Additional measures

A range of additional measures (using both quantitative and qualitative methods) will be collected for the purposes of answering questions about the potential mediation of treatment effects, referrals to other health professionals and utilisation of other services, barriers and facilitators to implementing the intervention, fidelity of the training provided, long-term implementation needs and economic analyses (Table 2). These analyses will not be used to measure treatment effectiveness. Specific instruments used include:

**Table 2:** Summary of other data collected from study patient participants. Baseline survey collected at 0 months

Outcome	Measurement tool	Collected at
Mediator measures		
Physical activity levels	Physical Activity Scale for the Elderly (PASE)	0, 6 and 12 months
Fear of movement	Brief Fear of Movement Scale (BFMS)	0, 6 and 12 months
Pain catastrophising	Pain Catastrophising Scale (PCS)	0, 6 and 12 months
Pain coping skills	Coping Strategies Questionnaire (CSQ)	0, 6 and 12 months
Ability to manage their condition	Effective Consumer Scale (EC17)	0, 6 and 12 months
Perception of their OA	Brief Illness Perception Questionnaire (B-IPQ)	0, 6 and 12 months
Arthritis self-efficacy	Arthritis Self-Efficacy Scale (ASES)	0, 6 and 12 months
Other patient collected data		
Post GP visit questionnaire	Custom questionnaire	Immediately after GP visit, ~ 2–6 weeks
Quality of OA care delivered during study	Osteoarthritis Quality Care Indicator (OA-QI)	6 and 12 months
Patient activation or self-management attitudes	Patient Activation Measure (PAM)	0 months
Treatment expectation	Custom questionnaire	0 and 6 months
Willingness for surgery	Custom questionnaire	0, 6 and 12 months
Participation in study components	Custom questionnaire	0, 6 and 12 months
Intervention adherence questions	Custom questionnaire	6 and 12 months
Adverse events	Custom questionnaire	0, 6 and 12 months

- Change in physical activity levels measured with the *Physical Activity Scale for the Elderly* (PASE), at 6 and 12 months. The PASE is a 10-item questionnaire used to measure both the frequency and type of recreational and occupational physical activity undertaken by participants over the previous seven days. Higher scores indicate greater levels of physical activity. The PASE was developed and validated in samples of older adults ≥ 55 years of age and has been used in many OA clinical trials [60].

b) Change in patient’s fear of movement and activity measured with the *Brief Fear of Movement Scale for Osteoarthritis* [61] at 6 and 12 months. The questionnaire consists of six statements and patients are asked to indicate how much they agree or disagree with each statement. The four response options range in value from 1 (strongly disagree) to 4 (strongly agree).

c) Change in patient’s pain catastrophising measured with the *Pain Catastrophizing Scale* (PCS) [62] at 6 and 12 months. The instrument is widely used in both clinical practice and research. The PCS is a 13-item instrument designed to quantify an individual’s pain experience and measure catastrophic thinking related to pain. Patients will be asked to indicate the degree to which they have the above thoughts and feelings when they are experiencing pain on a five point response scale ranging from 0 (not at all) to 4 (all the time). A total score is obtained (0–52), and can also be divided into three subscales that assess rumination, magnification and helplessness.

d) Change in patient’s pain coping skills at 6 and 12 months. This is measured using the *Coping Strategies Questionnaire* (CSQ) [63] at 6 and 12 months. The CSQ can be used to measure how often a patient uses six cognitive and behavioural pain coping strategies to manage pain (diverting attention, re-interpreting pain sensations, coping self-statements, ignoring pain sensations, praying and hoping, and increasing activity level). We will use the coping attempts subscale comprising 17 questions. Items are measured on a 7-point Likert scale (where 0 = never uses coping skills and 7 = always uses coping skills). Higher scores indicate greater coping skills. Based on prior factor analyses of this instrument [63] participant’s responses will be converted to scores on the Coping Attempts factor of the CSQ. The CSQ has demonstrated sensitivity to change from treatment in samples of people with chronic pain as well as good internal consistency and construct validity.

e) Change in patient’s ability to manage their condition at 6 and 12 months: The *Effective Consumer Scale* (EC17) will be used to determine how effective people are at dealing with their chronic condition and how well they make decisions about their health care [64]. This 17-item instrument has been validated in patients with arthritis diseases and comprises five domains looking at i) use of health information, ii) clarifying personal priorities, iii) communicating with others iv) negotiating roles and taking control, and v) deciding to take action. It is measured on a five-point scale with responses ranging from 0 (never) to 4 (always).

f) Change in patient’s perceptions of their OA at 6 and 12 months. The *Brief Illness Perception Questionnaire (B-IPQ)* [65] will be used to assess changes in the patient’s perception of their condition. The B-IPQ is an eight-item instrument that measures the cognitive perceptions with respect to a condition on an ordinal scale (0–10). The areas examined are: i) consequences, ii) timeline, iii) personal control, iv) treatment control, v) identity for describing the condition and symptoms, vi) coherence, and vii) concern and emotions. The maximal score is 80, with higher scores reflecting more negative perceptions.
g) Change in patient self-efficacy will be measured using the short form eight item *Arthritis Self-Efficacy Scale* (ASES) at 6 and 12 months [66]. It will be administered at baseline, 6 and 12 months. The 8-item short form is a validated version of the original version, and was designed to take less time for trial participants to complete. The participant is asked how certain they are that they can do the following tasks at the present time. The instrument includes two items on pain, four items on other symptoms, and two items that relate to preventing pain and fatigue from interfering with daily activities. Each question is ranked on a scale of 1–10 ranging from 1 “very uncertain” to 10 “very certain”. The total score is the mean of the eight items.

#### Economic evaluation

An economic evaluation will be conducted from the health care system perspective. We will assess cost-effectiveness of the intervention analysing a range of outcomes including incremental cost per extra person with a clinically significant improvement in pain (measured as 1.8 point reduction on the 0–10 pain score) and per quality-adjusted life years (QALYs) gained for the intervention group compared to the control group at 12 months. QALYs will be calculated based on utility scores using the AQoL-8D at baseline, and 12 months. We will compare the differences in health care usage and utility gains on the AQoL-8D over 12 months between intervention and control groups (adjusting for any difference in the baseline values between the groups). We will also compare changes in productivity lost from baseline to 12 months between the intervention and control groups.

### Data collection, management and analysis

#### Data collection and management

Study data will be primarily collected and managed using the REDCap tool hosted at the University of Sydney. REDCap is a secure, web-based application designed to support data capture for research studies [36]. Where hard copy questionnaires have been completed, they will be transcribed into REDCap by the research staff and the original scanned and uploaded to REDCap. We will collect MBS and PBS data from the Australian Commonwealth Department of Human Services (DHS) at the completion of the trial. Hard copy consent forms will be scanned and stored on a University of Sydney or University of Melbourne server, and the original form locked in a filing cabinet until it is sent to the DHS at the end of the trial. Study data, including MBS and PBS consent forms, will be retained for 15 years after which time it will be destroyed.

### Statistical methods

#### Sample size estimation, power and justification

Primary endpoints are at the patient level and are defined as changes in knee pain and function at 12 months. We wish to detect an effect size of 0.30 (moderate) [67]. Our sample size accounts for clustering effects due to people treated within the same GP practice. Assuming a minimum of 2 GPs per practice, 13 patients per GP practice recruited, with a coefficient of variation in practice size of 0.5, an intra-cluster correlation of 0.05 and up to 20% attrition of both GPs and patients, a total of 44 general practices and 572 patients will be required to detect the effect size with 80% power (two-sided significance level of 5%).

#### Statistical analysis

GP practices and individual participants will be analysed according to their randomised group, using intention-to-treat. Those practices that fail to recruit any patients or withdraw from the study prior to collecting data from any patients will be excluded from the analysis [68]. Descriptive statistics at the participant and GP practice level will be presented to allow comparison of treatment groups at baseline. Analyses will be conducted at the participant level. For continuous outcome measures, differences in mean change (baseline minus follow-up) will be compared between groups using generalised estimating equations (GEEs) to account for within-practice correlation with exchangeable correlation, robust standard errors, and adjusting for the baseline value of the outcome variable and stratification variables. For binary outcomes, GEEs will be fitted using a logit link function assuming an exchangeable correlation structure and robust variance estimation, adjusting for stratification variables, with results presented as odds ratios. To aid interpretation, risk differences will also be calculated using marginal probabilities [69]. Multiple imputation will be applied to missing participant-level data from GP practices which do not withdraw from the study before data collection and that recruit at least one patient [70].

An independent Data Safety and Monitoring Board (DSMB) will meet periodically to monitor the quality of trial data and the safety of patients. The DSMB may recommend continuing the trial or modifying the trial, or stopping the trial early. A recommendation to stop the trial may only be made to protect the safety of trial participants if there is clear evidence of a clinically important harmful effect.

### Timelines

The study application for funding was approved by the National Health and Medical Research Council (NHMRC) in May 2016, and funding commenced in January 2017. A pilot study to inform the main trial was undertaken from May to December 2017. Recruitment and training of the PARTNER CST occurred between May and October 2017. Recruitment for the RCT commenced in March 2018 and is due for completion in February 2019. The trial is due for completion in March 2020 after the 12 months data collection is complete.

### Adverse events

The risks for participants involved in this study are minimal. There will be multiple mechanisms for identifying serious adverse events (SAEs) that occur during the trial. Firstly, if the CST become aware of any SAE, they will inform the patient’s GP for follow-up and will advise the Chief Investigator and Trial Coordinator as soon as possible. Secondly, if a patient cannot be contacted over a four week period or at the end of the trial, the trial staff will contact the GP to confirm if the participant is still alive or has had any serious medical issues that have restricted their participation. Finally, the follow-up surveys and the health diary will ask patients to self-report any adverse events they may have experienced during the course of the trial. All SAEs will be reported to the approving HREC’s.

#### Future use of data

Information collected for this study may be used in future projects or submitted to a public database so that other researchers can access it and use it. At this stage, it is not known what these other projects will involve. We will seek ethical approval before using the information in these future projects, and any identifying information will be removed. All participants (general practices, GPs and patients), will be advised of the potential for future use of their de-identified data in the Patient Information Statement and Consent Forms.

## Discussion

Given knee OA is one of the most prevalent and disabling chronic diseases, even small reductions in ineffective practices with small improvements in care may lead to marked cost savings and reductions in the individual and societal burden of the disease. If the PARTNER intervention rationale is correct and implementation is successful, the results will have major significance for Australia with potential implications internationally, including potential relevance to low- and middle-income settings in the context of remotely-delivered care. The intervention is intentionally complex and targets both GPs and patients. By improving the capacity of GPs to offer their patients support for behavioural change through the CST, we hope to ensure uptake and maintenance of exercise and weight loss and better satisfaction with the care they are provided. We also hope to improve access to high-quality self-management support and behavioural counselling for patients, and by so doing improve adherence to effective conservative non-drug treatment including exercise and weight loss, thus reducing the OA burden.

We have clear intentions for subsequent implementation by planning to ensure the intervention is acceptable, feasible, aligned with current Australian models of care and to assess its cost-effectiveness. We will assess the barriers and facilitators to more widespread implementation and work closely with stakeholders including consumers, government and insurers to ensure policy recommendations stem from this work if found to be effective and cost-effective.

## Abbreviations

ABS: Australian Bureau of Statistics
ADL: Activities of Daily Living
AQoL-8D: Assessment of Quality of Life Instrument
ASES: Arthritis Self-Efficacy Scale
BFMS: Brief Fear of Movement Scale
B-IPQ: Brief Illness Perception Questionnaire
BMI: Body Mass Index
CBT: Cognitive Behavioural Therapy
cdmNet: Chronic Disease Management Network software
CSQ: Pain Coping Strategies Questionnaire
CST: PARTNER Care Support Team
DHS: Commonwealth of Australia Department of Human Services
DSMB: Data Safety Monitoring Board
EC-17: Effective Consumer Scale
GPs: General Medical Practitioners
HREC: Human Research Ethics Committee
HWFL: Osteoarthritis Healthy Weight For Life Program
KOOS: Knee injury and Osteoarthritis Outcome Score
MBS: Medical Benefits Scheme
Medicare: Universal health care system funded by the Commonwealth Government of Australia
NHMRC: Australian National Health and Medical Research Council
NICE: National Institute for Health and Clinical Excellence
OA: Osteoarthritis
OA-QI: Osteoarthritis Quality Indicator Questionnaire
OARSI: Osteoarthritis Research Society International
PAM: Patient Activation Measure
PARTNER: Optimising primary care management of knee osteoarthritis trial
PASE: Physical Activity Scale for the Elderly
PBS: Pharmaceuticals Benefits Scheme
PCS: Pain Catastrophising Scale
PHQ-9: Patient Health Questionnaire
PHReNet: New South Wales Primary Health Care Research Network
PROMIS™: Patient-Reported Outcomes Measurement Information System
QALYs: Quality-adjusted Life Years
QI&CPD: Quality Improvement and Continuing Professional Development
QoL: Quality of Life
RACGP: Royal Australian College of General Practitioners
RCT: Randomised Controlled Trial
REDCap: Research Electronic Data Capture software
SAE: Serious Adverse Event
UNSW Australia: University of New South Wales, Sydney, Australia
VicReN: Victorian Primary Care Practice-Based Research Network
WPAI:OA: Work Productivity and Activity Impairment Questionnaire: Osteoarthritis of the Knee
