# Correction to: Effectiveness of a new model of primary care management on knee pain and function in patients with knee osteoarthritis: Protocol for THE PARTNER STUDY

**DOI:** 10.1186/s12891-018-2362-6

**Published:** 2018-12-20

**Authors:** D. J. Hunter, R. S. Hinman, J. L. Bowden, T. Egerton, A. M. Briggs, S. J. Bunker, J. Kasza, A. B. Forbes, S. D. French, M. Pirotta, D. J. Schofield, N. A. Zwar, K. L. Bennell, the PARTNER Study Team

**Affiliations:** 1 Institute of Bone and Joint Research, Kolling Institute, University of Sydney, Sydney, Australia; 2 Department of Rheumatology, Royal North Shore Hospital, Sydney, NSW Australia; 3 Centre for Health, Exercise and Sports Medicine, Department of Physiotherapy, The University of Melbourne, Melbourne, Victoria Australia; 4 School of Physiotherapy and Exercise Science, Curtin University, Perth, WA Australia; 5 Department of Physiotherapy, The University of Melbourne, Melbourne, Victoria Australia; 6 Biostatistics Unit, School of Public Health and Preventive Medicine, Monash University, Melbourne, Victoria Australia; 7 School of Rehabilitation Therapy, Queen’s University, Kingston, Ontario Canada; 8 Department of Chiropractic, Faculty of Science and Engineering, Macquarie University, Sydney, NSW Australia; 9 Department of General Practice, The University of Melbourne, Melbourne, Victoria Australia; 10 Department of Economics, Faculty of Business and Economics, Macquarie University, Sydney, Australia; 11 School of Public Health and Community Medicine, University of New South Wales, Sydney, NSW Australia; 12 School of Medicine, University of Wollongong, Wollongong, NSW Australia; 13 Centre for Health, Exercise and Sports Medicine, Department of Physiotherapy, The University of Melbourne, Melbourne, Victoria Australia

**Erratum: BMC Musculoskeletal Disorders**

**https://doi.org/10.1186/s12891-018-2048-0**

After the publication of this protocol [1], our collaborator Prima Health solutions advised us of their intent to withdraw from the study. Their primary role was to provide remotely delivered weight-loss services (via the Healthy Weight for Life program) to eligible participants in the intervention group. These services were partly provided as in-kind and partly funded through the study. We have received ethical approval from the University of Sydney to replace the Healthy Weight for Life program with the Commonwealth Scientific and Industrial Research Organisation’s (CSIRO) Total Wellbeing Diet. The amended *weight loss advice and support* paragraph of the manuscript is outlined below*.* All changes to the protocol were made and approved before starting the trial and were prospectively changed on our trial registration (ACTRN12617001595303).

***Amended weight loss advice and support paragraph:*** If the patient has a BMI ≥27 kg/m^2^, the patient will be offered the option of participating in the remotely-delivered weight loss program. The Australian Commonwealth Scientific and Industrial Research Organisation’s (CSIRO) “Total Wellbeing Diet” is based on an evidence-based weight management strategy that utilises a structured, nutritionally balanced eating plan designed to be incorporated into a balanced lifestyle program [2, 3]. The program is a 12- week, low glycaemic index, high protein, healthy eating program with online support and tracking tools, meal plans and educational resources on a healthy diet. It is delivered by SP Health (http://www.sphealth.com/) on behalf of the CSIRO. After completion of the 12-week program, patients may elect to continue the basic program for an additional 12-weeks. Patients who elect to undertake the online weight-loss program will continue to be supported by the PARTNER Care Support Team throughout their time on the weight-loss program. This program will be undertaken in conjunction with the PARTNER exercise program and educational resources on healthy lifestyle change.
